# The importance of tumor studies in the initial evaluation for Lynch syndrome: a comparison of MMR positive and negative patients

**DOI:** 10.1186/1897-4287-9-S1-P34

**Published:** 2011-03-10

**Authors:** Devki S Saraiya, Y Nancy You, Robert L Askew, Thuy M Vu, Patrick M Lynch, Miguel Rodriguez-Bigas

**Affiliations:** 1 Gastroenterology, Hepatology & Nutrition, UT M.D. Anderson Cancer Center, Houston, TX 77030, USA; 2 Surgical Oncology, UT M.D. Anderson Cancer Center, Houston, TX 77030, USA

## Background

Hereditary non-polyposis colorectal cancer or Lynch syndrome (LS) is characterized by mismatch repair (MMR) loss of function. The aim of this study was to compare the clinical characteristics and family histories of MMR mutation-positive and mutation-negative patients with tumor studies suggestive for LS.

## Methods

Patients with loss of MLH1/MSH2 on immunohistochemistry (IHC) or with microsatellite instability (MSI)-high tumors were identified in our institutional LS database (February 1992–June 2010). Patients who subsequently underwent MLH1/MSH2 mutation analysis were reviewed. Patients with no identifiable MLH1 germline mutation were excluded if MLH1 promoter methylation was present or not assessed. Patients with variants of uncertain significance were also excluded. Demographics, clinical characteristics, mutational testing, and family histories of patients were analyzed.

## Results

Of 92 patients with informative tumor studies who underwent germline mutation testing with unequivocal results, 61 (58.7%) were mutation-positive and 31 (29.8%) were mutation-negative. The mutation detection rate for MLH1 and MSH2 was 51.2% and 78.4%, respectively. A significant difference (p=0.006) in the proportion of MLH1 and MSH2 defects was found between mutation groups (Table 1).

**Table 1:** 

	Gene		CRC			
	**MLH1**	**MSH2**	**Diagnosed**	**Age at diagnosis (SD)**	**Proximal location**	**Synchronous / metachronous**

**Mutation Positive n=61**	21 (34.4%)	40 (65.6%)	52 (85.2%)	43.4 (11.5)	25 (48.1%)	17 (32.7%)
**Mutation Negative n=31**	20 (64.5%)	11 (35.5%)	24 (77.4%)	50.0 (11.7)	15 (62.5%)	5 (20.8%)

Colorectal cancer (CRC) was diagnosed in 76 patients (82.6%; Table 1). Mutation-positive patients had a younger mean age of CRC diagnosis (43.4 vs. 50.0, p=0.011). Endometrial and other LS cancers were diagnosed in 36.0% and 29.5% of mutation-positive patients and 27.8% and 16.1% of mutation-negative patients, respectively. Clinical criteria were more sensitive in identifying MMR-positive than MMR-negative families (Amsterdam I: 42.6% vs. 9.7%, p=0.001; Amsterdam II: 60.7% vs. 12.9%, p<0.001; Bethesda: 93.4% vs. 67.7%, p=0.004). Mutation-positive patients meeting at least one of these criteria on average had more than twice as many affected relatives (3.7 vs. 1.5, p=<0.001).

## Discussion

In this population of patients with presumptive LS diagnoses based upon tumor studies, mutation-negative patients had significant personal cancer histories concerning for LS with slightly later ages of initial LS cancer and CRC diagnosis. However, their family histories were significantly less suggestive as nearly one-third of patients were missed by Bethesda criteria and over 85% by Amsterdam I/II criteria. This suggests that both established clinical criteria and germline mutation analysis may fail to detect a significant number of patients with presumed LS and supports the use of routine MSI/IHC analyses to identify these otherwise undetectable high-risk families.

